# Study of dynamic brain function in irritable bowel syndrome via Hidden Markov Modeling

**DOI:** 10.3389/fnins.2024.1515540

**Published:** 2025-01-13

**Authors:** Chuan Jing, Tianci Liu, Qingzhou Li, Chuan Zhang, Baijintao Sun, Xuezhao Yang, Yutao You, Jixin Liu, Hanfeng Yang

**Affiliations:** ^1^Department of Radiology, Affiliated Hospital of North Sichuan Medical College, Nanchong, China; ^2^Center for Brain Imaging, School of Life Science and Technology, Xidian University, Xi’an, China

**Keywords:** irritable bowel syndrome, Hidden Markov Model, resting-state functional magnetic resonance imaging, dynamic brain functioning, brain state

## Abstract

**Background and purpose:**

Irritable bowel syndrome (IBS) is a common bowel-brain interaction disorder whose pathogenesis is unclear. Many studies have investigated abnormal changes in brain function in IBS patients. In this study, we analyzed the dynamic changes in brain function in IBS patients using a Hidden Markov Model (HMM).

**Methods:**

Resting-state functional magnetic resonance imaging (rs-fMRI) data and the clinical characteristics of 35 patients with IBS and 31 healthy controls (HCs) were collected. The rs-fMRI data of all participants were analyzed using HMM to identify recurrent brain activity states that evolve over time during the resting state. Additionally, the temporal properties of these HMM states and their correlations with clinical scale scores were examined.

**Result:**

This study utilized the Hidden Markov Model (HMM) method to identify six distinct HMM states. Significant differences in fractional occupancy (FO) and lifetime (LT) were observed in states 5 and 6 between the IBS and HCs. The state transition probabilities differed between IBS and HCs, with an increased probability of transitioning from state 2 to state 6 in IBS patients. The reconfiguration of HMM states over time scales in IBS patients was associated with abnormal activity in the default mode network (DMN), sensorimotor network (SMN), and cingulo-opercular network (CON).

**Conclusion:**

This study offers novel insights into the dynamic reorganization of brain activity patterns in IBS and elucidates potential links between these patterns and IBS-related emotional regulation and symptom experience, thereby contributing to a deeper understanding of the neural mechanisms underlying IBS.

## 1 Introduction

Irritable bowel syndrome (IBS) is a very common bowel-brain interaction disorder, characterized by changes in bowel frequency and stool properties associated with abdominal pain (Mearin et al., 2016). It is often accompanied by psychological distress, such as anxiety and depression (Simrén et al., 2019), but without detectable definitive organic lesions. The global prevalence of IBS is about 11%, the gastrointestinal symptoms and psychiatric comorbidities of IBS seriously affect the quality of life of patients and consequently incur high healthcare costs (Ford, 2020). Owing to the complexity of the pathogenesis of IBS, which involves genetic, environmental, psychological, and physiological factors, the exact pathogenesis of IBS has not been fully elucidated (Holtmann et al., 2016). With the development of the field of neurogastroenterology in recent years, the concept of the gut-brain axis, which refers to a bidirectional communication system between the brain and the gut involving neural, endocrine, and immune pathways, has been recognized (Hillestad et al., 2022). Several studies have suggested that the brain plays a significant role in the development and maintenance of symptoms in IBS patients (Chen N. et al., 2021; Li et al., 2024). Therefore, studying brain function can help elucidate the pathogenesis of IBS.

Functional magnetic resonance imaging (fMRI) is an effective tool for studying brain function. Studies on IBS have shown that rectal distension can cause an increase in the activation of the anterior cingulate cortex, insula, and ventromedial prefrontal regions of the salience network (SN) in IBS patients (Hall et al., 2010). Elsenbruch et al. reported that during rectal distension, IBS patients exhibited abnormal activation in brain regions involved in the regulation of emotion, particularly in the insula, amygdala, and prefrontal cortex (PFC) (Elsenbruch et al., 2010). Additionally, another experiment on rectal distension revealed excessive activation in the regions associated with emotion and attention in IBS patients (Hong et al., 2016). Some researchers reported that IBS patients have lower activation in the medial prefrontal cortex (mPFC), posterior cingulate cortex (PCC), and bilateral inferior parietal cortex within the default mode network (DMN) during rectal distension (Qi et al., 2016). A study (Chen X. F. et al., 2021) reported abnormal spontaneous brain activity and dysfunctional connectivity in regions of the prefrontal-limbic-mesencephalic circuitry associated with pain modulation and emotional arousal in IBS patients compared to healthy controls (HCs). Some researchers (Li et al., 2021) reported that IBS patients with depressive symptoms presented functional connectivity (FC) abnormalities in frontal limbic and sensory-motor network-associated brain regions, particularly the insula and supplementary motor area (SMA), with FC abnormalities. Another study (Sun Q. et al., 2024) revealed that patients with IBS and patients with major depressive disorder (MDD) presented impaired corticostriatal functional connectivity. Multiple functional networks in the brains of IBS patients have undergone abnormal changes, primarily involving the SN, DMN, sensorimotor network (SMN), and executive control network (ECN) (Nisticò et al., 2022), which may be closely related to the processing of visceral afferent signals, emotional arousal, pain modulation, and cognitive regulation. However, these functional MRI studies were conducted based on the fact that functional connectivity in the brain remains static and unchanged during fMRI data acquisition. Some studies have shown that functional connectivity in the brain is not static but is a dynamic process that produces variations over time (Chang and Glover, 2010). This finding implies that the assumption that the brain is static during fMRI data acquisition ignores the variability of the brain over time and greatly simplifies the functional connectivity of the brain (Allen et al., 2014). Studies on various disorders, such as vestibular migraine (VM) (Xiong et al., 2024), (MDD) (Sun S. et al., 2024), and schizophrenia (González-Peñas et al., 2024), have shown that determining the changes in brain network dynamics can help us increase our understanding of the disease pathogenesis.

The sliding window method is a commonly used approach for studying brain network dynamics, however, the sliding window method has several limitations, firstly, the optimal window size needs to be predefined and selected, an extremely short window length can lead to a reduction in the observed sample data and increase the risk of observing false fluctuations, whereas a very long window length can interfere with the temporal changes in the region of interest (Hutchison et al., 2013), in addition, the sliding window method lacks sensitivity to transient changes in time series (Vidaurre et al., 2021). Another approach to studying brain network dynamics is the Hidden Markov model (HMM) (Ahrends et al., 2022). The advantage of HMM in the study of brain functional networks lies in its ability to simultaneously capture the brain’s activity states and functional connectivity patterns, HMM defines the complex dynamic activities of the brain as discrete spatial states, with each state representing a specific activation pattern and functional connectivity pattern of brain activity (Vidaurre et al., 2017). This not only helps to identify different brain activity states, but also helps to reveal the temporal evolution patterns of these states. Compared with traditional sliding window methods, the time scale of HMM does not need to be predetermined but is determined adaptively by the data (Stevner et al., 2019), furthermore, HMM can precisely capture the dynamic reorganization of the brain network at the smallest time scale (Quinn et al., 2018), effectively overcoming the limitations of the sliding window method. The dynamic transformation between brain networks is not entirely random, but rather repeats over time (Vidaurre et al., 2017), HMM can effectively capture these brain states that repeatedly appear over time, helping to reveal the flexibility and stability of different brain functions. Therefore, HMM not only provides a detailed description of brain activity, but also captures the time-varying characteristics of brain networks, providing a new perspective for deeply understanding the dynamic properties of the brain.

In this study, HMM was used to analyze rs-fMRI data from IBS patients and HCs, to identify different brain states in IBS patients and HCs. By analyzing the temporal properties of HMM states and brain activation areas between the two groups, we explored the dynamic changes in brain activity of IBS patients. Based on previous research, we hypothesize that the brain activity states and functional connectivity patterns of IBS patients have changed, with differences in temporal attributes that may be related to emotional regulation and symptom experience.

## 2 Materials and methods

### 2.1 Participants

All experimental procedures in this study followed the principles of the Declaration of Helsinki. The research program was approved by the Ethics Committee of the Affiliated Hospital of North Sichuan Medical College. All participants voluntarily signed an informed consent form before participating in the experiment.

Between June 2023 and July 2024, 35 IBS patients (21 males and 14 females) were recruited from the outpatient clinic of the Department of Gastroenterology at the Affiliated Hospital of North Sichuan Medical College. Moreover, 31 healthy people were recruited as HCs (19 males and 12 females) in the hospital. To ensure that the participants met the experimental criteria, they were diagnosed by a professional gastroenterologist and underwent physical examination, blood biochemistry examination, gastrointestinal endoscopy, etc. Patients with IBS were diagnosed based on the Rome IV criteria and excluded from other diseases that may cause similar symptoms.

The inclusion criteria for patients with IBS were as follows: (1) right-handed, male and female; (2) meeting the Rome IV diagnostic criteria (Drossman, 2016); (3) no antidepressants or medications with therapeutic effects on symptoms of IBS were used within 3 months.

The exclusion criteria for patients with IBS were as follows: (1) those with a history of gastrointestinal surgery or organic bowel disease; (2) those with current or past mental and nervous system diseases; (3) patients with severe acute/chronic organic diseases such as those of the heart, kidney, and liver; (4) patients with other chronic pain (such as headache, low back pain, etc.); (5) those who were administered antidepressants and medications for treating IBS symptoms within the past 3 months; (6) MRI contraindications, including claustrophobia and metal implants or devices in the body.

The inclusion criterion for HCs was right-handed, age-matched, and sex-matched patients with IBS. The HC exclusion criteria were the same as the IBS exclusion criteria.

### 2.2 Clinical symptoms and psychological assessment

Before the patients underwent MRI, basic clinical information on their sex, age, and education level was collected. Additionally, to measure the severity of symptoms, severity of depression and anxiety, and quality of life of patients with IBS, each patient with IBS was evaluated using the following scales: the IBS Severity Scoring System (IBS-SSS) (Francis et al., 1997), the Patient Health Questionnaire Depression Scale (PHQ-9) (Kroenke et al., 2001), the Pain Anxiety Symptom Scale (PASS) (Rogers et al., 2020), and the IBS-Specific Quality of Life Scale (IBS-QOL) (Patrick et al., 1998). The IBS-SSS is used to measure the severity of IBS. It consists of a five-part scale with a total score of 500 points, with scores of 75–175 indicating mild, 176–300 indicating moderate, and more than 300 indicating severe. The PHQ-9 consists of nine items and is widely used in the clinical assessment of depressive symptoms, with scores of 0–4 indicating the absence of depression and scores of 5, 10, 15, and 20 indicating mild, moderate, moderately severe, and severe depression, respectively. The PASS is a scale specifically designed to assess symptoms of anxiety associated with pain. It consists of 20 items ranging from “never” to “frequently” on a scale of 0–5, with higher scores indicating higher levels of pain anxiety and fear. The IBS-QOL, which consists of 34 items, is used to measure health-related quality of life in patients with IBS.

### 2.3 Magnetic resonance imaging data acquisition

All participants underwent MRI scanning using a Siemens Skyra 3.0 T scanner and a standard 20-channel head and neck coil. The scan covered the whole brain. Foam pads were used to immobilize the head to reduce head movement. Earplugs were worn to reduce external stimuli, and participants were instructed to keep their eyes closed but stay awake, keep their bodies still and relaxed, and breathe calmly during the scan. The rs-fMRI data and 3D-T1 data were acquired for each participant. The rs-fMRI data were acquired via a gradient echo-planar imaging (EPI) sequence. The scanning parameters were as follows: TR: 2,000 ms, TE: 30 ms, number of scanned layers: 200, layer thickness: 3 mm, FOV: 240 mm × 240 mm, intralaminar resolution: 3 mm × 3 mm. The 3D-T1 data were obtained via a fast spin echo sequence. The following scanning parameters were used: phase encoding direction A → P, TR: 2,240 ms, TE: 3.73 ms, number of scanned layers: 192, layer thickness: 1 mm, FOV: 256 mm × 256 mm, intralayer resolution: 1 mm × 1 mm, and scanning time: 4:46.

### 2.4 Magnetic resonance imaging data preprocessing

The software package DPABI (V8.1) was used to preprocess the rs-fMRI data in MATLAB. First, the MRI data were converted from DICOM format to the NIFTI format. Second, the first 10 volumes of the functional images were deleted to ensure that the image had a uniform signal, and then the sampling time error was eliminated by layer time correction. Through head motion correction, the data with head motion exceeding 3 mm and a rotation angle exceeding 3° were excluded. Next, the 3D-T1 structural image was matched with the functional image and segmented into gray matter, white matter, and cerebrospinal fluid, and then registered into the Montreal Neurological Institute (MNI) space for resampling, the resampling resolution was 3 mm × 3 mm × 3 mm, and a Gaussian kernel with a half-maximum (FWHM) of 6 mm was used for spatial smoothing to reduce registration inaccuracy. The Friston 24 parameter model was used to adjust the head motion parameters, and the white matter and cerebrospinal fluid signals were used as covariates for regression. The global signal regression was not used (Hahamy et al., 2014; Saad et al., 2012). Finally, the time series of the rs-fMRI data was bandpass filtered (0.01–0.08 Hz) to improve the signal-to-noise ratio.

### 2.5 Constructing Hidden Markov Models

**Figure 1:**
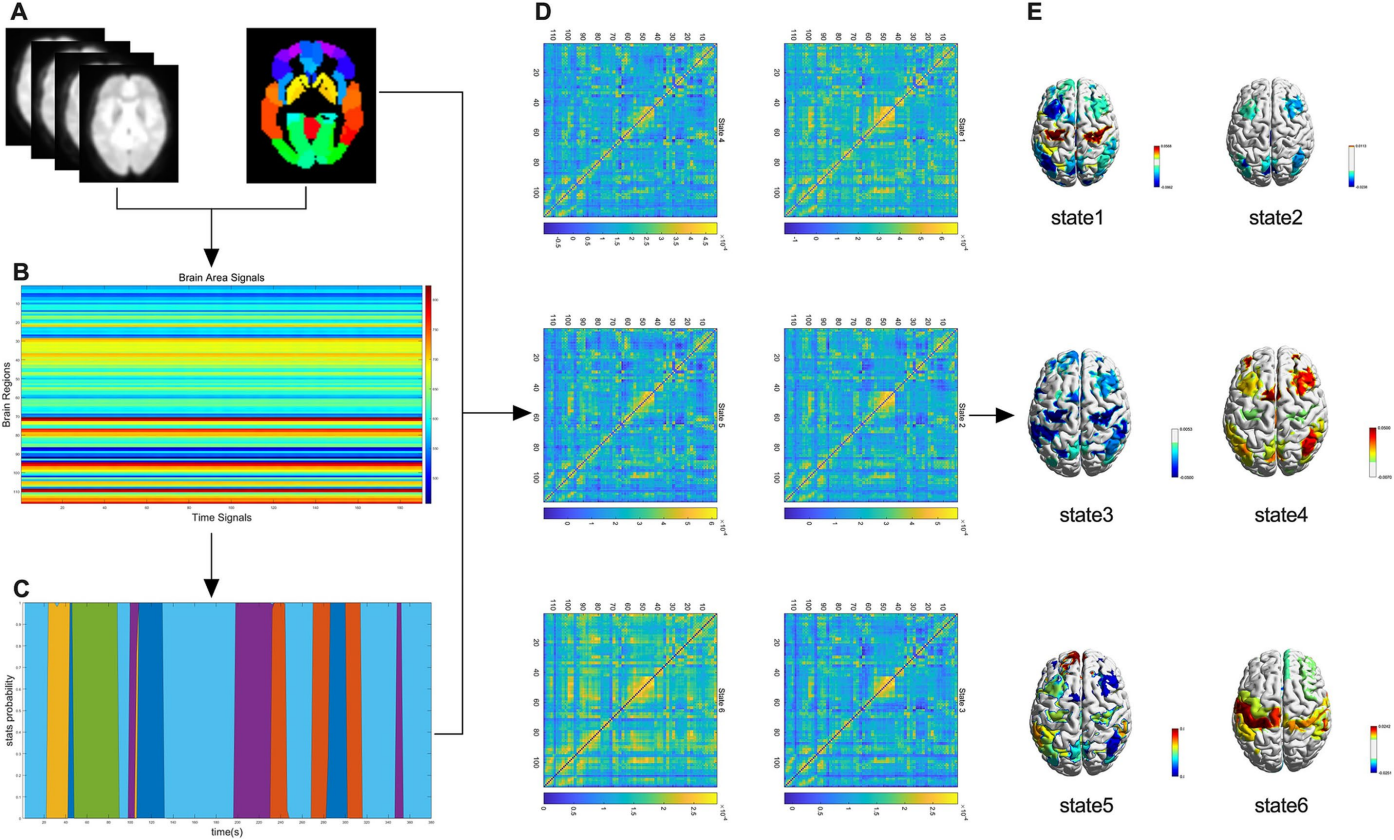
A flowchart of the HMM study. **(A)** Whole-brain regional delineation of each participant was divided into 116 regions of interest according to the automated anatomical labeling (AAL) atlas. **(B)** Temporal data for 116 regions × 190 time points were obtained for each participant. **(C)** Probability of occurrence of the state at different time points. **(D)** Six HMM states were identified after the data were evaluated. **(E)** Brain-averaged activation maps for each HMM state are shown.

The HMM is a Markov process involving hidden states, and the variations in brain activity across time scales can be explained by a finite number of hidden states (Javaheripour et al., 2023). First, the whole brain regions of all participants were divided into 116 regions of interest (ROIs) based on the automated anatomical labeling (AAL) atlas (Figure 1A). Then, each participant obtained time data for 116 regions × 190 time points (Figure 1B) and the occurrence probability of states at different time points (Figure 1C), and used it to construct the HMM. Based on previous studies (Moretto et al., 2022), we performed iterative calculations between 2 and 15 states and found that the HMM reached the minimum free energy state when the number of states was 6; thus six HMM states were selected (Figure 1D). Finally, the average activation map of the brain was generated according to HMM state (Figure 1E).

### 2.6 Temporal properties of HMMs

Based on the obtained HMM states, the following metrics that can respond to the temporal dynamics of the brain were analyzed. (1) Fractional occupancy (FO) refers to the duration of a particular state throughout the time series; FO can help us understand how the states are distributed among each other over a period. (2) Lifetime (LT): This refers to the duration of a state before a state transition occurs, i.e., how long the current state has been maintained before transitioning to another state. LT reflects the stability of the state; a longer LT may indicate that the state is more stable, whereas a shorter LT may indicate that the state transitions are more frequent. (3) The switching rate (SR) describes the frequency of transitions between different states; the state switching rate refers to the frequency of transfer from one state to another within a unit of time, and this metric reflects the speed of dynamic changes between states. (4) The state transfer probability, which is the core of the HMM, represents the possibility of transferring from one state to another.

### 2.7 Statistical analysis

All statistical analyzes were conducted using SPSS 27.0. Continuous variables are shown as mean ± standard deviation (SD), whereas categorical variables are shown as counts. The Shapiro–Wilk (SW) test was conducted to determine whether the data followed a normal distribution. THE independent samples *t*-test was conducted to compare age differences between the two groups, and the chi-square test was conducted to compare gender differences between the groups. A nonparametric permutation test was conducted with 5,000 permutations to compare the temporal properties of HMM states between the IBS and HC groups, and the differences were considered to be statistically significant at *p* < 0.05 after FDR correction. Finally, spearman correlation analysis was performed to evaluate the association between depressive and pain anxiety, as well as the association between HMM state time attributes and IBS-SSS, PHQ-9, and PASS scores, the differences were considered to be statistically significant at *p* < 0.05 after FDR correction.

## 3 Results

### 3.1 Demographics and clinical measures

In this study, 35 IBS patients (14 females and 21 males) and 31 HCs (12 females and 19 males) were selected. The demographic and clinical characteristics of the IBS patients are listed in Table 1. No significant differences were found between IBS patients and HCs in terms of age, sex, or education level (all *p* > 0.05).

**Table 1:** Demographic and clinical data.

	IBS (*n* = 35)	HC (*n* = 31)	*p*-value
Gender (male/female)	21/14	19/12	0.558^a^
Age (year)	47.85 ± 7.79	45.12 ± 11.45	0.258^b^
Education level (year)	14.20 ± 2.37	14.80 ± 2.67	0.341^b^
IBS-SSS	223.71 ± 50.11	–	–
PHQ-9	5.17 ± 3.97	–	–
PASS	29.42 ± 16.22	–	–
IBS-QOL	50.94 ± 11.44	–	–

IBS, irritable bowel syndrome; HC, healthy control. Values are mean ± SD or N. IBS-SSS, IBS Severity Scoring System; PHQ-9, Patient Health Questionnaire depression scale; PASS, Pain Anxiety Symptom Scale; IBS-QOL, IBS-Quality of Life.

a Statistical differences were obtained by using chi-square test.

b Statistical differences were obtained by using independent samples *t*-test.

### 3.2 Differences in the temporal properties of HMM states

In this study, the HMM was applied to analyze the rs-fMRI data of IBS patients and HCs. Six states were identified, and their temporal properties were compared. The main results are described below.

#### 3.2.1 Fractional occupancy

**Figure 2:**
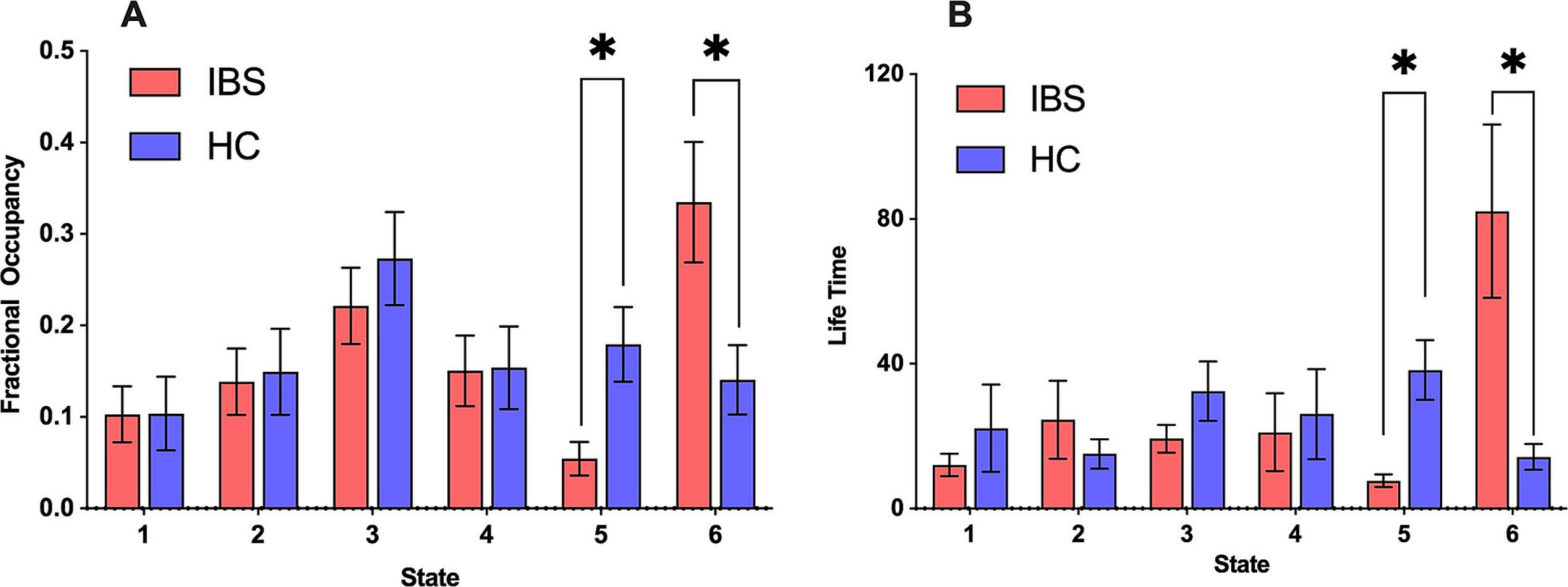
FO and LT for different HMM states of HCs and IBS patients. **(A)** Comparison of FO between IBS patients and HCs in each state. **(B)** Comparison of LT between IBS patients and HCs in each state. Red represents IBS, and blue represents HC. All time features were compared by conducting a nonparametric permutation test with 5,000 permutations, *p < 0.05.

The IBS patients had a lower FO in state 5 than HCs, whereas in state 6, IBS patients had a significantly greater FO (Figure 2A); the FOs of the remaining states did not differ significantly between HCs and IBS patients.

#### 3.2.2 Lifetime

In IBS patients, the changes in LT were similar to those of FO, i.e., IBS patients had shorter LTs in state 5, whereas, in state 6, the LT of IBS patients was significantly longer. This suggested that state 6 was more stable in IBS patients and state 5 was more stable in HCs (Figure 2B). The LTs of the remaining states were not significantly different between HCs and IBS patients.

#### 3.2.3 State switching rate and state transfer probability

**Figure 3:**
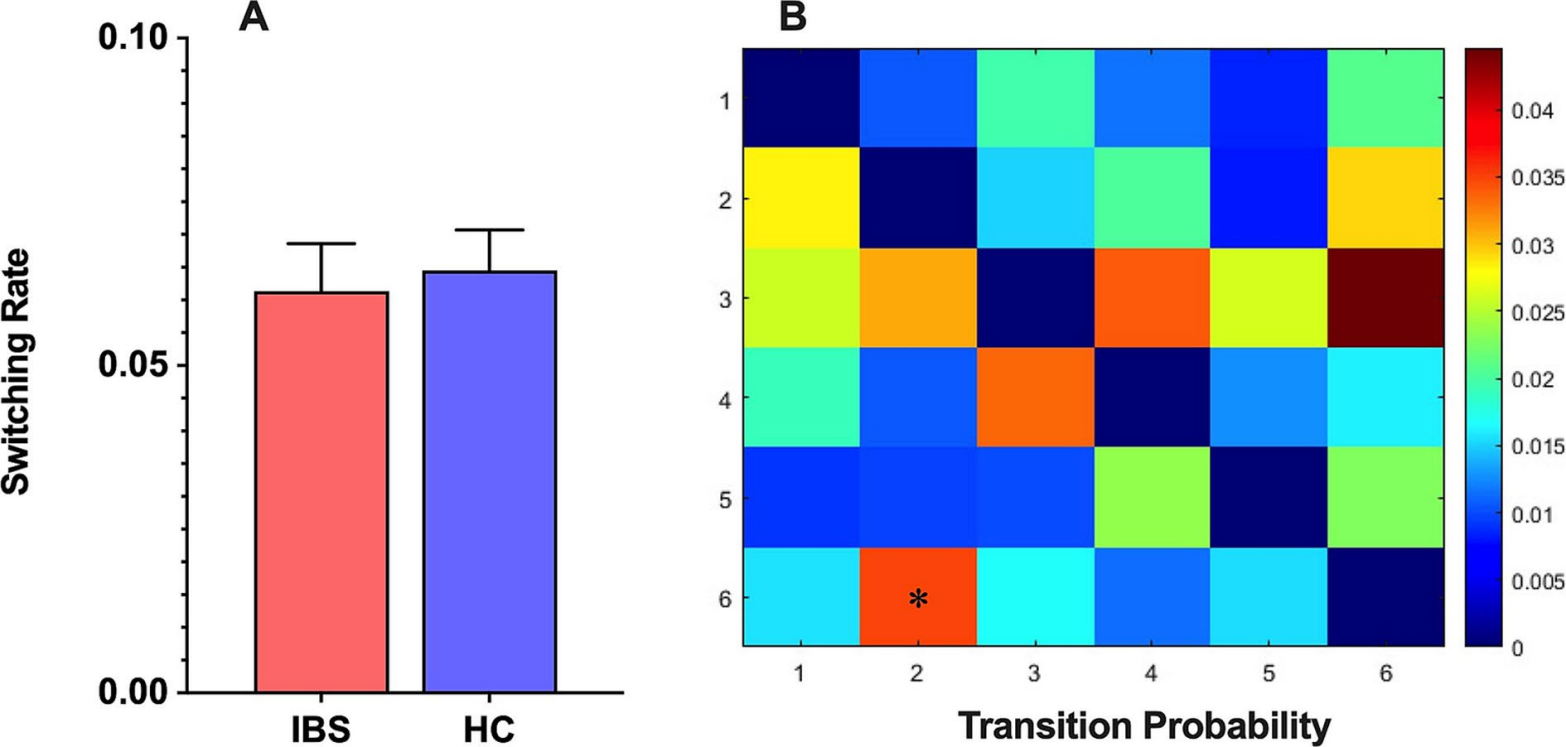
**(A)** The HMM state switching rate between IBS patients and HCs. **(B)** The HMM state transition probabilities between IBS patients and HCs and differences between the two groups were compared using a nonparametric permutation test with 5,000 permutations, *p < 0.05.

The analysis of the state switching rate revealed no significant difference in the switching rate between the IBS patients and HCs (Figure 3A). This finding suggested that the IBS patients and HCs had similar brain state switching frequencies during the scanning process. Further analysis revealed a significant difference in the switching probability among the different HMM states between the two populations, with a significantly greater probability of transfer from HMM state 2 to state 6 in IBS patients than in HCs (Figure 3B).

### 3.3 Results of spearman correlation analysis

**Figure 4:**
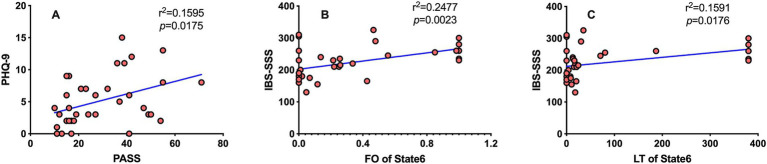
The results of Spearman correlation analysis. Spearman correlation analysis was performed, and the results were considered to be statistically significant at *p* < 0.05 after FDR correction. **(A)** The PHQ-9 is positively correlated with the PASS scores (*r*^2^ = 0.1595, *p* = 0.0175). **(B)** FO of HMM status 6 was positively correlated with the IBS-SSS score (*r*^2^ = 0.2477, *p* = 0.0069). **(C)** LT of HMM status 6 was positively correlated with the IBS-SSS score (*r*^2^ = 0.1591, *p* = 0.0264).

The PHQ-9 and PASS scores of IBS patients are positively correlated (Figure 4A), the FO and LT of status 6 were positively correlated with the severity of IBS (Figures 4B,C) but not with depression or pain anxiety. The temporal attributes of the other states are not correlated with IBS-SSS, PHQ-9, and PASS scores.

### 3.4 Functional connectivity of HMM states

**Figure 5:**
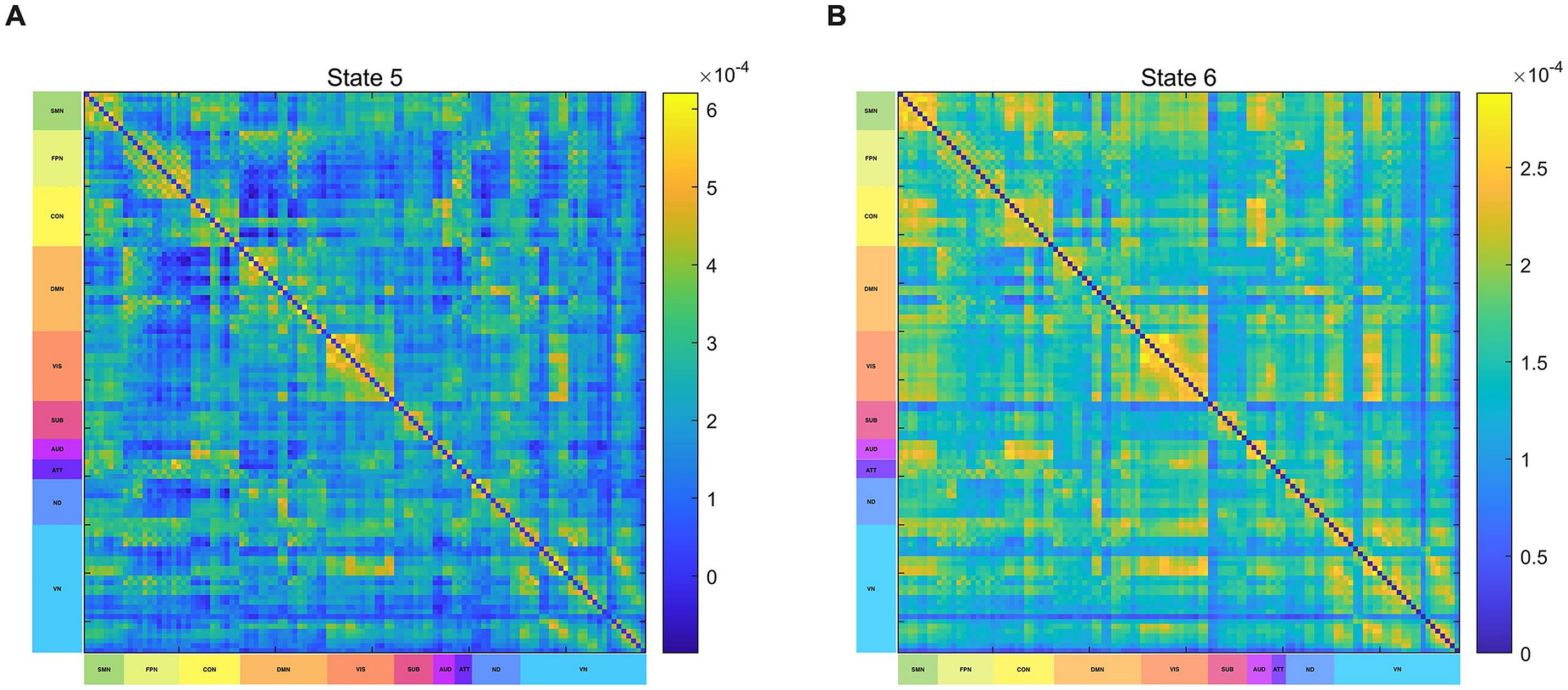
Functional connectivity of HMM states. **(A)** Brain functional connectivity in State 5. **(B)** Brain functional connectivity in State 6. SMN, sensorimotor network; VIS, visual network; AUD, auditory network; DMN, default-mode network; FPN, frontoparietal network; CON, cingulo-opercular network; CN, cerebellar network; SUB, subcortical network; ATT, attention network; ND, not defined.

The HMM state differences between IBS patients and HCs were reflected mainly in states 5 and 6. Based on previous research (Cao et al., 2020; Long et al., 2019), 116 brain regions have been assigned to the following brain networks: SMN, DMN, Frontoparietal Network (FPN), Cingulo-Opercular Network (CON), Subcortical Network (SBN), Visual Network (VIS), Auditory Network (AUD), Attention Network (ATT), and Cerebellar Network (CN). The primary characteristic of the FC in state 5 is the moderately strong intra-network connections within the FPN, CON, and DMN, while the inter-network connections between the DMN and CON are significantly weak (Figure 5A), In state 6, the primary characteristic is the significantly strong intra-network connections within the SMN and CON, while the inter-network connections between the DMN and CON are relatively weak (Figure 5B).

### 3.5 Brain activation maps of the HMM states

**Figure 6:**
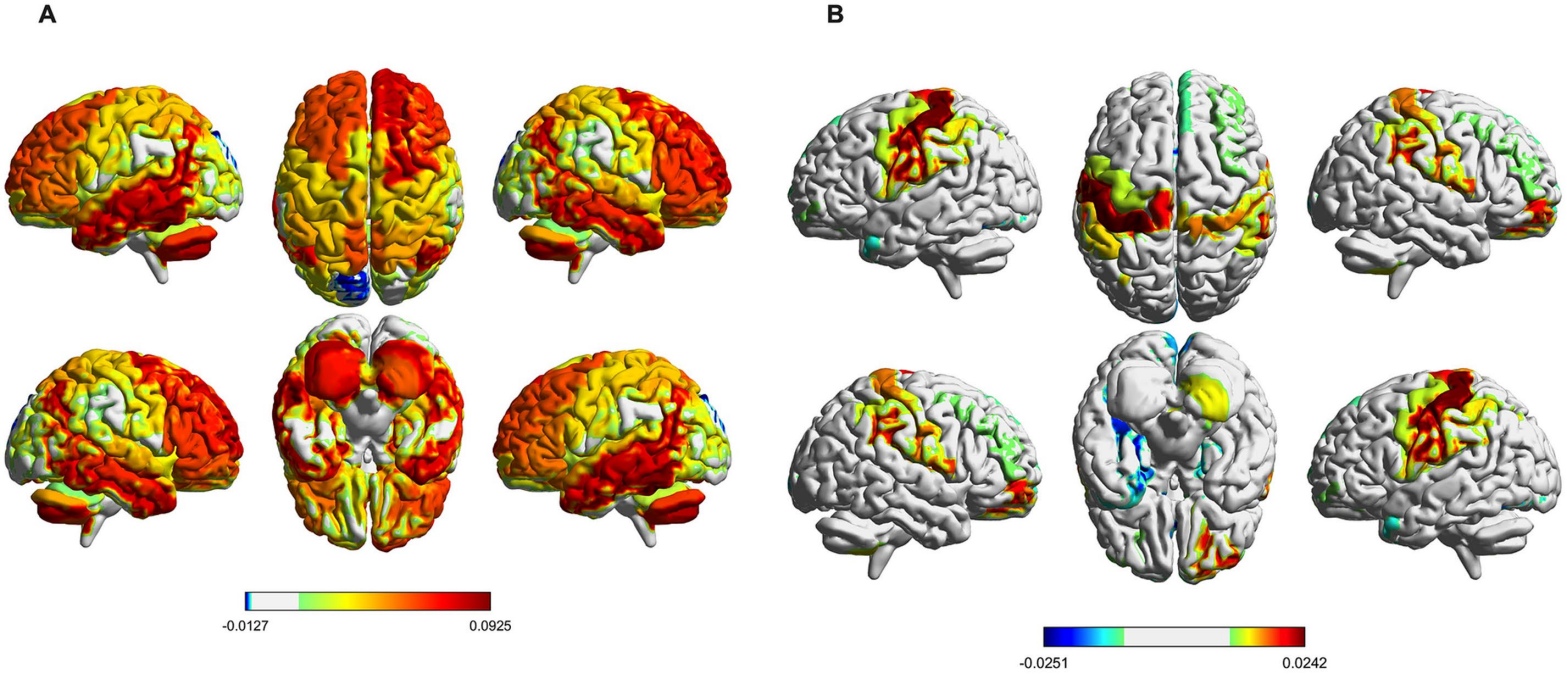
Spatial activation map of brain states. **(A)** Distribution of mean brain activation areas for state 5. **(B)** Distribution of mean brain activation areas for state 6.

The brain activation map in state 5 is primarily mediated by HC, and the regions with increased brain activation are mainly located in the right anterior cingulate with the paracingulate gyrus, right posterior cingulate gyrus, left inferior temporal gyrus, and cerebellum, the regions with decreased brain activation are primarily found in the left cuneate lobe and the left superior occipital gyrus (Figure 6A). The brain activation map in state 6 is primarily mediated by IBS, the regions with increased brain activation are mainly located in the right orbitofrontal middle gyrus and left postcentral gyrus, the regions with decreased brain activation are primarily found in the right anterior cingulate with the paracingulate gyrus, left parahippocampal gyrus, and lenticular nucleus (Figure 6B).

## 4 Discussion

In this study, the fMRI data was analyzed using HMM, and 6 HMM states were identified. By comparing the changes in the temporal properties of the HMM states of IBS and HC, we observed that dynamic brain activity in IBS exhibited abnormal changes and temporal reorganization. Additionally, the analysis of functional connectivity and average brain activation maps for each HMM state revealed specific and differential patterns of large-scale brain network activity in IBS. Compared to traditional resting-state functional connectivity and dynamic functional connectivity analyzes, HMM provided a more detailed description of temporal sequences.

HMM explores complex brain activity by analyzing the state of brain activity and the probability of state transition. In this study, six different HMM states were identified using HMM, and the temporal properties (including FO, LT, SR, and state transition probabilities) of each state were analyzed. We found that the brain network activity state in IBS undergoes temporal reconfiguration. Specifically, in state 5, IBS exhibits lower FO and LT, Similar temporal attribute differences have also been reported in HMM studies of MDD (Javaheripour et al., 2023). Anxiety and depression are the most common extra-intestinal symptoms in IBS patients. Correlation analysis between PHQ-9 and PASS scores indicates that pain anxiety can elevate the level of depression in IBS patients, suggesting the presence of emotional regulation disorders in this population. The PFC is a well-known brain region involved in emotion regulation and is often hypoactive in mood-disorder-like disorders (Pizzagalli and Roberts, 2022; Tillisch et al., 2011). In state 5, the lower FO and LT in IBS reflect the instability of brain functions associated with emotion regulation. The emotional regulation disorder in IBS patients may be linked to this state of instability. Our research findings offer new insights into understanding the frequent occurrence of depressive symptoms in IBS patients from a temporal perspective, which may help elucidate the potential neural mechanisms underlying emotional regulation disorders in IBS patients. Furthermore, IBS showed a significant increase in FO and LT in state 6, indicating that IBS has higher stability in this state. This suggests that state 6 may be a characteristic brain state of IBS. The results of the subsequent correlation analysis showed that FO and LT in state 6 were positively correlated with the severity score of IBS, so we hypothesize that the temporal difference in state 6 may be mediated by the severity of IBS symptoms. The typical symptoms of IBS include changes in the frequency and character of bowel movements associated with abdominal pain, which is a key part of the condition. Signals from abdominal pain travel along the gut-brain axis to the central nervous system of the brain, where the brain alters central control of visceral sensitization and pain perception via the neurotransmitters 5-hydroxytryptamine (5-HT) and norepinephrine (NA) (Hanna-Jairala and Drossman, 2024). The changes observed in state 6 in IBS may indicate dysfunction of the gut-brain axis and trigger neural remodeling in the brain. Gut-brain axis dysfunction plays an important role in IBS (Ford et al., 2024; Ray and Ghoshal, 2024). These findings provide evidence of IBS gut-brain axis dysfunction over time and offer potential biomarkers for assessing the impact of IBS symptom severity.

Brain activity is a complex dynamic process. The cognitive and regulatory functions of the brain are realized through transitions between different brain states (Ahrends et al., 2022). Understanding the transitions between these states is crucial for elucidating the plasticity of brain function in IBS patients. We analyzed the switching rates of the six HMM states and found no significant difference in the state switching rates between the IBS and HC groups, suggesting that IBS patients and HCs have similar brain network switching frequencies. However, a significant difference was found in the state shift probability between IBS patients and HCs, with a significant increase in the probability of IBS state 2 shifting to state 6, suggesting that the switching patterns of brain networks may have altered, and IBS patients tend to transition from state 2 to state 6.

In the HMM analysis, each state represents a specific brain function pattern, reflecting different configurations of brain network activity. The functional connectivity matrix of State 6 shows that the functional connections within the SMN and CON are in a strong connected state, while the functional connections between the DMN and CON are in a relatively weak connected state. The average brain activation maps show increased activation in the SMN and decreased activation in the DMN for IBS patients. The DMN is a brain network that has been extensively studied in IBS research. When attention is concentrated on introspective cognitive thinking activities, the activation of the DMN network increases, when attention is attracted by sensory stimuli such as pain, the activity of the DMN decreases (Menon, 2023). Qi et al. found that compared to healthy controls (HC), the functional connectivity of the Default Mode Network (DMN) was decreased in IBS patients (Qi et al., 2016). Another study discovered that after treatment with lidocaine, IBS patients experienced a significant reduction in pain sensation, and the connectivity within the DMN, including the middle temporal gyrus, angular gyrus, and inferior temporal gyrus, was enhanced (Letzen et al., 2013). In this study, the reduced activation of the DMN reflects the impairment of self-awareness and introspective thinking functions in IBS patients, further proving that the DMN plays an important role in the pathogenesis of IBS (Zhao et al., 2023). SMN network is responsible for the processing and regulation of visceral and somatic sensory information (Mayer et al., 2023). Previous studies have indicated structural and functional abnormalities in the SMN of IBS patients, in adolescent girls experiencing irritable bowel syndrome, enhanced functional connectivity within the SMN has been observed (Bhatt et al., 2019), Grinsvall et al. found that IBS patients have increased gray matter volume in the primary and secondary somatosensory cortices as well as subcortical regions, while there is a reduction in gray matter volume, surface area, and cortical thickness in the posterior insula and superior frontal gyrus (Grinsvall et al., 2021). In this study, the overactivation of SMN reflects the dysfunction of IBS patients in processing and regulating visceral pain sensation, this may be closely related to the severity of IBS patients’ symptoms and the degree of visceral hypersensitivity. The CON has complex dynamic interactions with multiple functional networks related to cognitive control. It is not only involved in regulating behavior and controlling movements but also in processing pain stimuli (Han et al., 2023). In this study, the abnormal connectivity patterns between the CON and the DMN and SMN may reveal the dysfunctions in cognitive control, emotional regulation, and pain perception in IBS patients. In summary, our research results reveal abnormal activity patterns in multiple brain networks of IBS patients, which could help further understand the neurological mechanisms behind IBS.

Although the HMM provided new insights in analyzing functional changes in IBS brain networks, the limitations of this study need to be considered. First, our sample size is small, which will affect the reliability of the study results and limit the generalization. Future studies should consider recruiting a wider range of participants to improve the reliability of the study results. Secondly, HMM assumes that states are mutually exclusive, meaning that only one brain state is activated at a specific point in time. Another assumption of HMM is that when the state at a certain point in time is known, the state at the next point in time can be inferred without needing to know the sequence of states prior to that point, which is referred to as short-range dependency. This is inconsistent with previous research on the brain’s long-range dependency, thus HMM has potential limitations in accurately characterizing brain states. Thirdly, the number of HMM states is a free parameter. Ideally, more states can provide more temporal information. The number of states determined in our study is the optimal number for resolving the current data. Lastly, in recent years, machine learning has played a significant role in studying brain function and exploring disease mechanisms (Zhang et al., 2020; Zong et al., 2024; Zuo et al., 2024). However, due to the heterogeneity in data collection and processing methods, machine learning has been less applied in neuroimaging studies of IBS. The powerful data processing capabilities of HMM can effectively identify brain states and transition patterns that differ between IBS and HC and provide temporal characteristics of brain network activity (such as FO, LT, and SR). These parameters can serve as input features for machine learning models to enhance the diagnostic and differential diagnostic efficacy of IBS. They can also predict changes in IBS symptoms and responses to treatment. Therefore, combining HMM with machine learning may help further understand the neurological mechanisms of IBS and assist clinicians in providing personalized treatment plans for IBS patients.

## 5 Conclusion

To summarize, we used HMM to investigate the complex brain activity in IBS and HC and identified six different brain states. Compared with HC, IBS patients showed significant differences in the temporal attributes of states 5 and 6. Additionally, IBS patients exhibited abnormal activation patterns and functional connectivity in multiple brain networks, which may be related to the patients’ emotional regulation and symptom experience.

## Data Availability

The raw data supporting the conclusions of this article will be made available by the authors, without undue reservation.
